# Hybrid surgical approach for a large schwannoma from the cervical esophagus to the upper thoracic esophagus: a case report

**DOI:** 10.1186/s44215-024-00171-5

**Published:** 2024-10-16

**Authors:** Masashi Nakagawa, Naoki Mori, Kohei Saisyo, Takehumi Yoshida, Taro Isobe, Hisamune Sakai, Toru Hisaka, Nobuya Ishibashi, Fumihiko Fujita

**Affiliations:** Department of Surgery, Kurume University School of Medicine, 67 Asahi Machi, Kurume City, Fukuoka 830-0011 Japan

**Keywords:** Esophageal schwannoma, Sternocleidomastoid muscle flap, Thoracoscopic surgery

## Abstract

**Background:**

Esophageal schwannoma is an extremely rare esophageal submucosal tumor. We report a case of a hybrid surgery for a large esophageal schwannoma that had extended from the cervical to the upper thoracic esophagus by using thoracoscopic and cervical approaches.

**Case presentation:**

A 58-year-old male was referred to our hospital for further examination and treatment of dysphagia and weight loss over the past 6 months. Upper gastrointestinal endoscopy revealed a 5.7-cm submucosal tumor from the cervical esophagus to the upper thoracic esophagus. The submucosal tumor was diagnosed as esophageal schwannoma by endoscopic ultrasound-guided fine-needle aspiration biopsy (EUS-FNA). Contrast-enhanced CT showed that the tumor had not invaded surrounding organs. Since the tumor extended from the cervical esophagus to the upper thoracic esophagus, we decided that it should be resected by not only the cervical but also the thoracoscopic approach. In operation, the patient was first placed in the prone position, and a thoracoscopic dissection of the upper thoracic esophagus containing the tumor was performed from the surrounding area. After changing the patient’s position from prone to supine, a cervical skin incision was performed, and we underwent the tumor enucleation. After enucleation, the esophageal wall was thinned, so the right sternocleidomastoid muscle was used to reinforce the esophageal wall. The tumor size of the specimen was 60 × 52 × 42 mm. The postoperative course was uneventful, and the patient was discharged on the 22nd day after surgery.

**Conclusions:**

Enucleation of a large esophageal schwannoma from the cervical to the upper thoracic esophagus could be safely performed using both thoracoscopic and cervical approaches. The sternocleidomastoid muscle flap is useful in the occasion considering stenosis by muscular layer suture.

## Background

Schwannoma, the most common type of neurogenic tumor, occurs in the head and neck region and the extremities. Gastrointestinal tumors are rare, especially esophageal schwannoma is very rare [1]. The diagnosis of esophageal schwannoma may not be confirmed by biopsy, and resection is recommended for definitive diagnosis. However, the surgical technique varies from each institution depending on the location and size of the tumor. Malignant schwannoma has rarely been reported [2, 3], but the term “schwannoma” itself refers to a benign, slow-growing tumor, and the majority of these tumors are benign [1]. Therefore, esophageal schwannoma is compatible with minimally invasive surgery. We report a minimally invasive thoracoscopic and cervical approach to enucleation of a schwannoma from cervical esophagus to upper thoracic esophagus.

## Case presentation

**Fig. 1:**
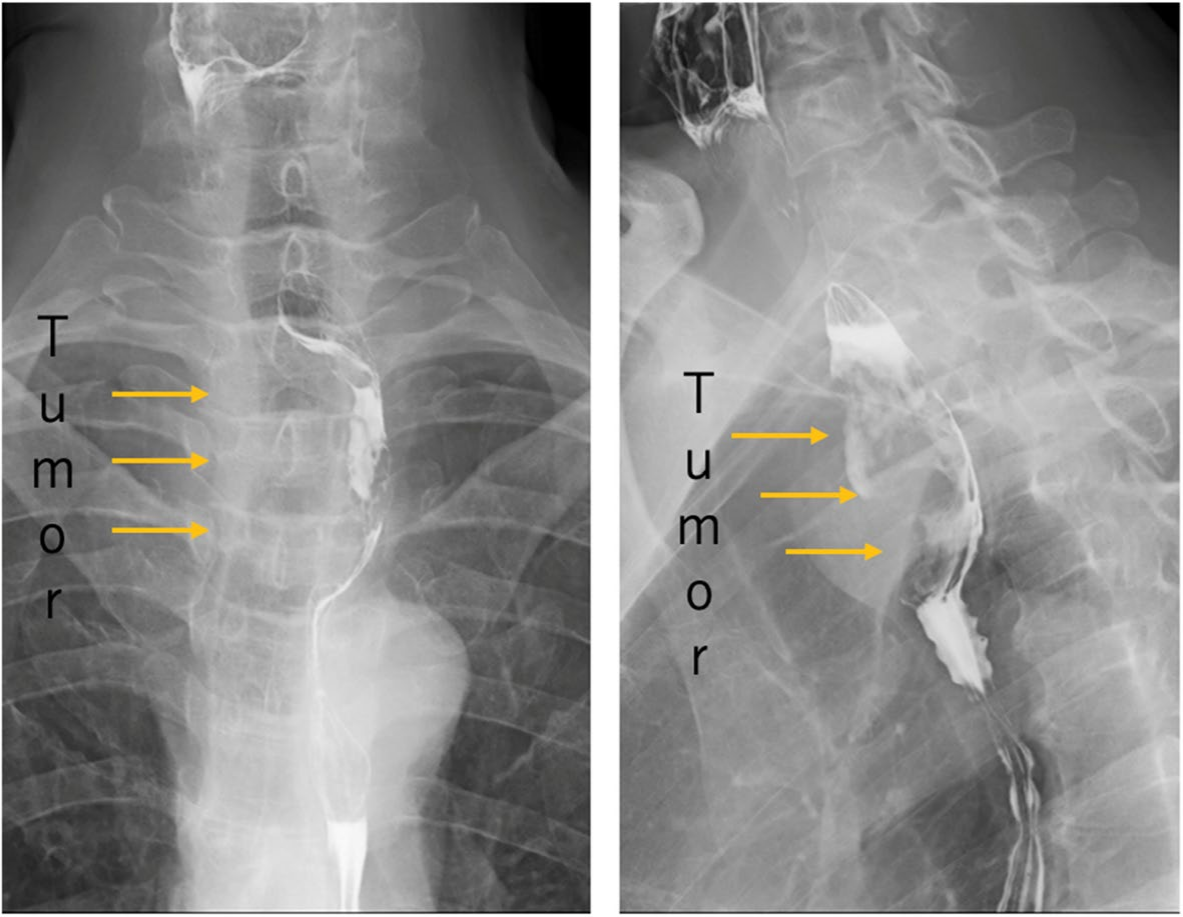
Upper gastrointestinal fluoroscopy revealed a 5.7-cm tumor spanning from the cervical to the upper thoracic esophagus with a smooth mucosal surface

**Fig. 2:**
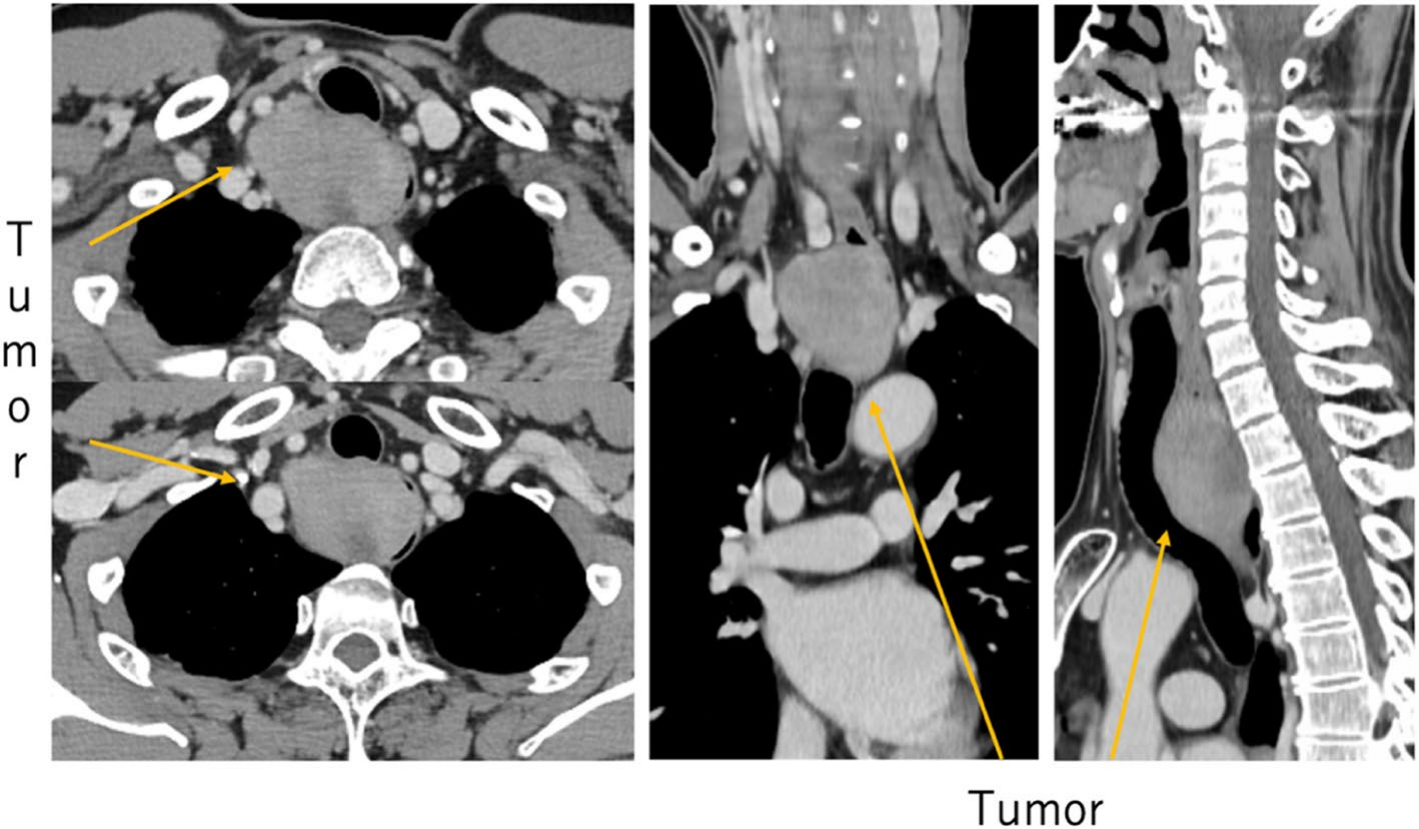
A contrast-enhanced CT scan revealed a uniformly enhanced 5 × 3.5 × 5.4 cm tumor without invasion to other organs or enlarged surrounding lymph nodes

**Fig. 3:**
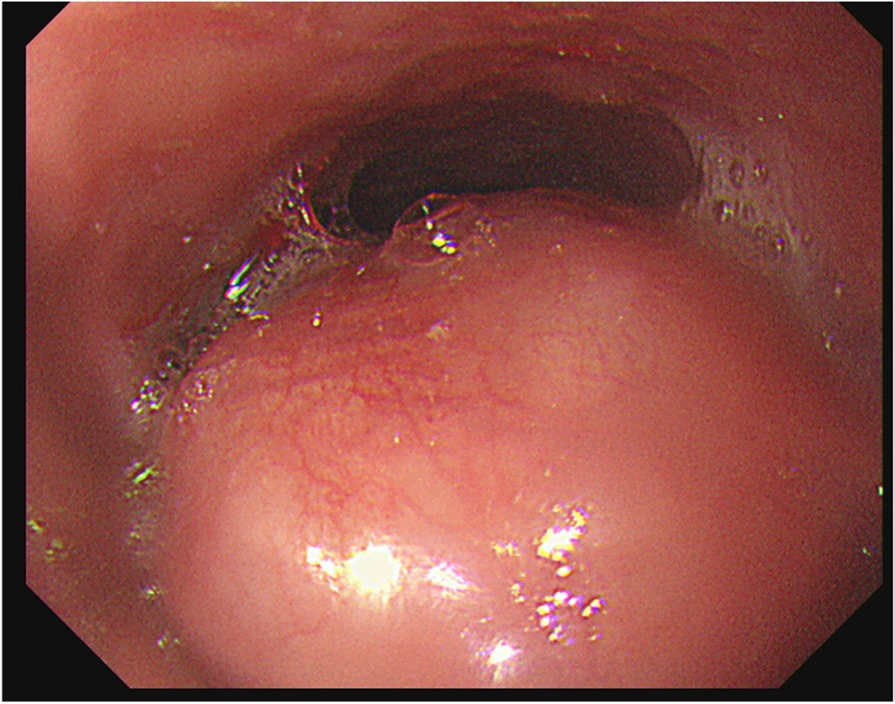
EGD findings: a submucosal tumor with a smooth surface was found 22 cm from the incisors. The oral esophagus did not dilate

**Fig. 4:**
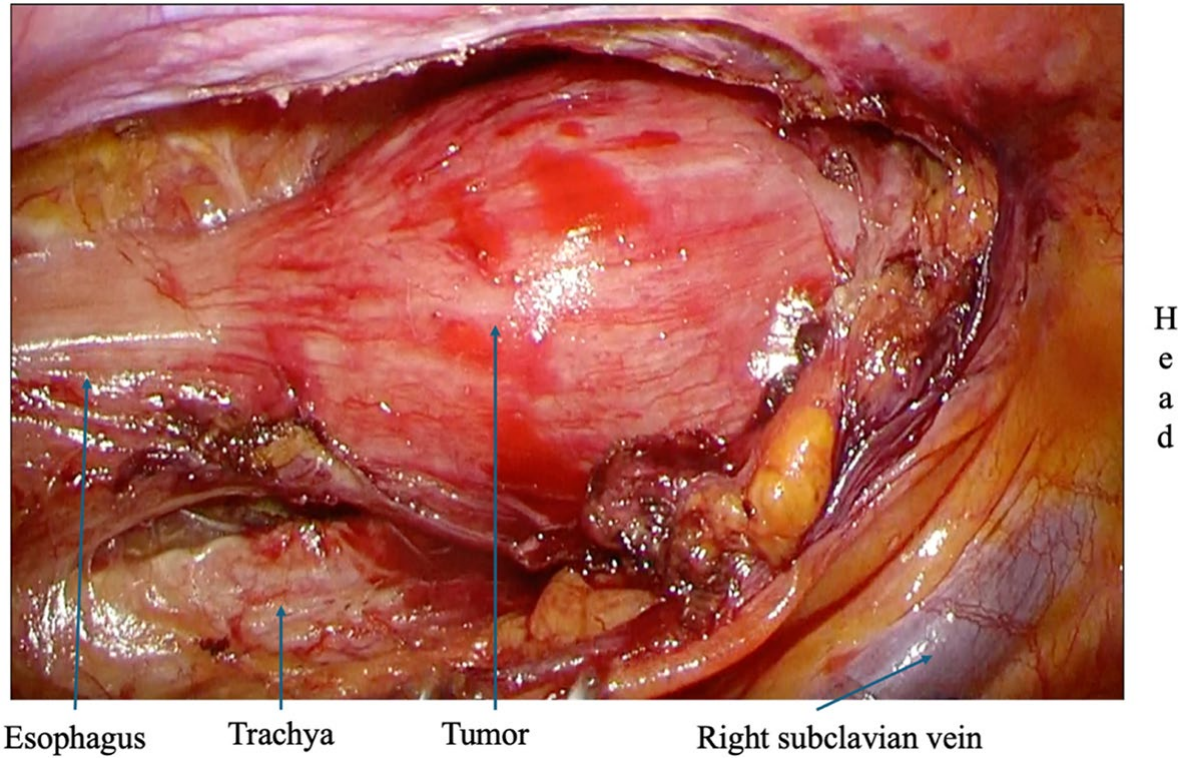
Operative findings: the tumor extended to the cervical from the upper thoracic esophagus. It was difficult to dissect the cranial side of the tumor

**Fig. 5:**
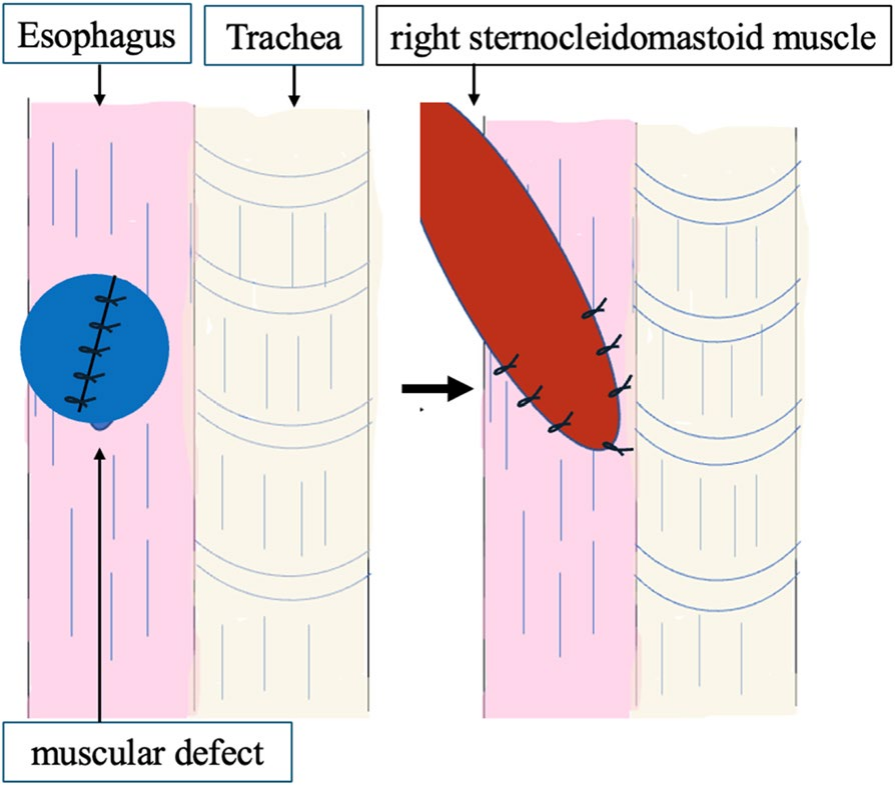
Operative finding: 1-cm muscular defect was observed. The fault was covered with a right sternocleidomastoid muscle flap

**Fig. 6:**
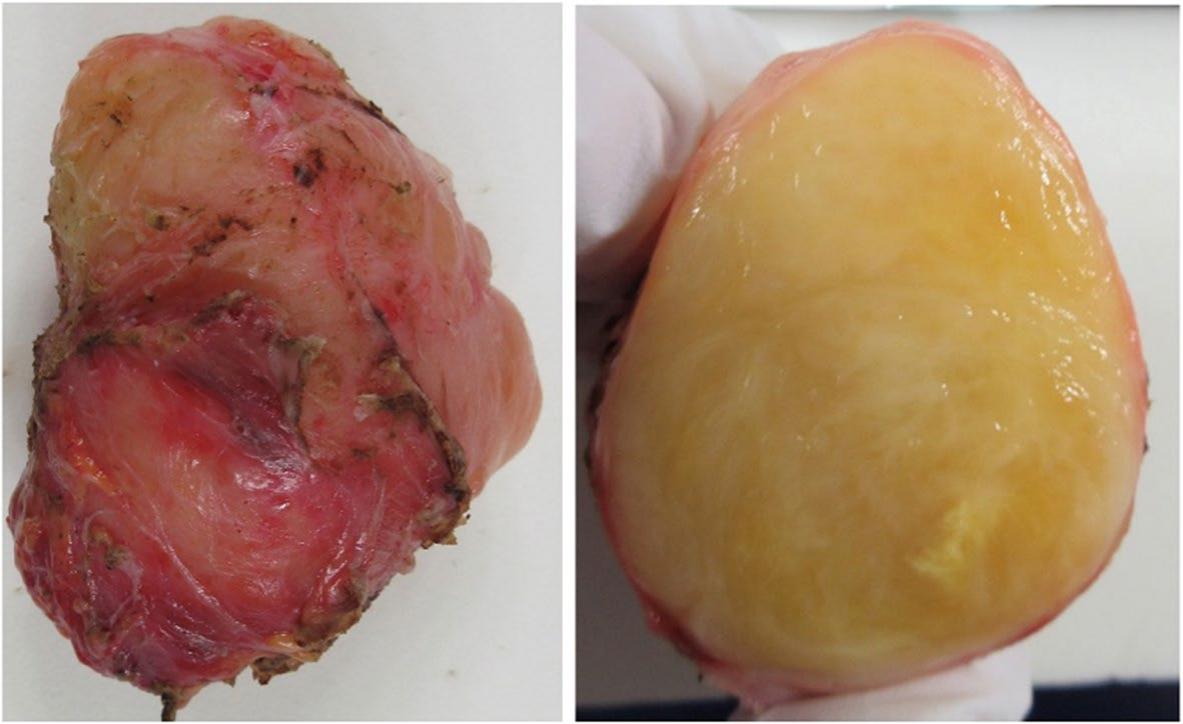
The resected specimen was yellow, hard, and elastic and measured 6 × 5.2 × 4.2 cm

**Fig. 7:**
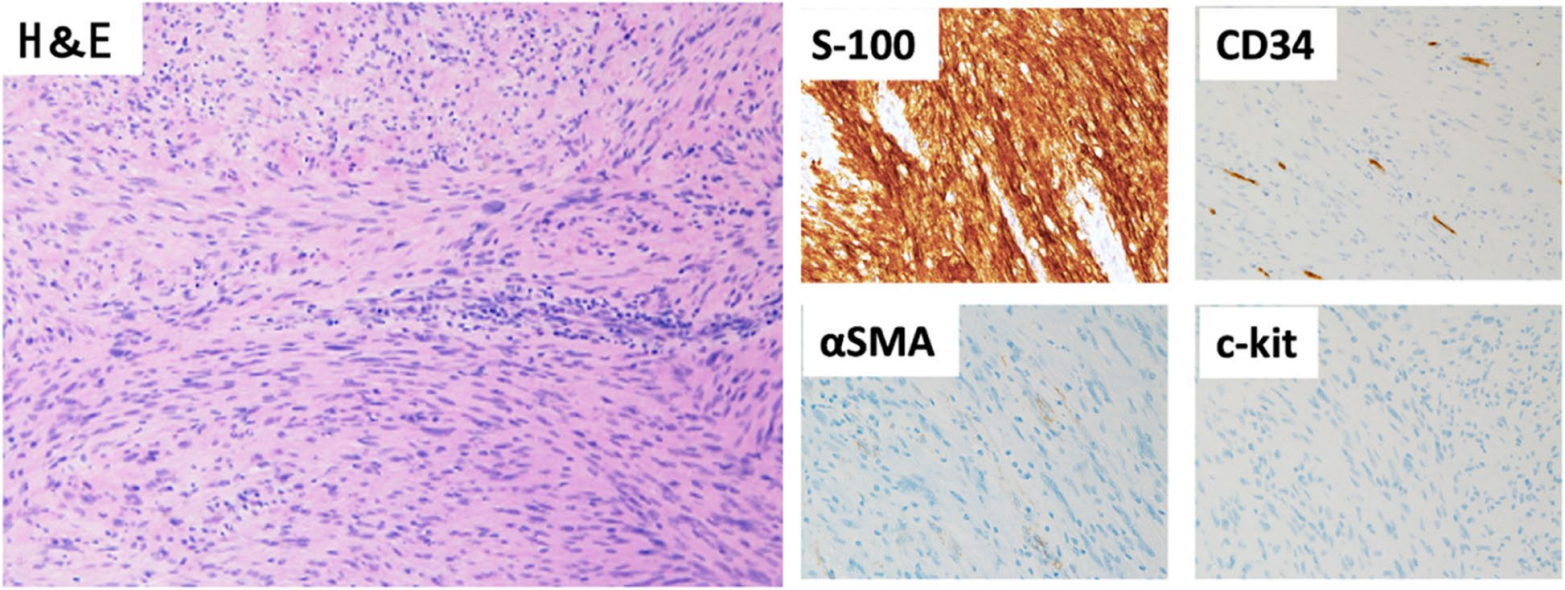
Immunohistochemistry results CD34 ( −), c-kit ( −), desmin ( −), and S100 ( +) confirmed a benign esophageal schwannoma

**Fig. 8:**
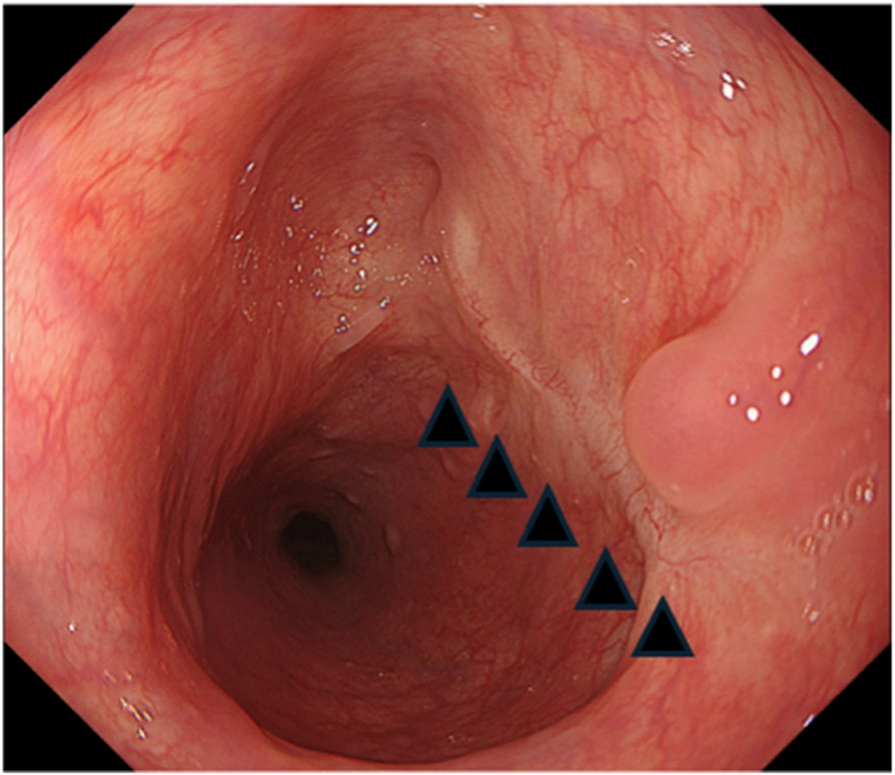
After the surgery, an endoscopy conducted 2 years later showed no signs of narrowing or ischemic changes

The patient was a 58-year-old male who consulted a local physician due to dysphagia and weight loss over the past 6 months. An esophagogastroduodenoscopy (EGDs) revealed a submucosal tumor from cervical to upper thoracic esophagus, leading to a referral to the Department of Gastroenterology at Kurume University. His medical history included bronchial asthma, for which he was receiving inhalation therapy. He occasionally consumed alcohol and had no smoking history. Blood tests showed no elevation of tumor markers. Upper gastrointestinal fluoroscopy revealed a 5.7-cm tumor spanning from the cervical to the upper thoracic esophagus with a smooth mucosal surface and no mediastinal shift (Fig. 1). A cervico-thoraco-abdominal contrast-enhanced CT scan revealed a uniformly enhanced 5 × 3.5 × 5.4 cm tumor without with no evidence of invasion to other organs nor enlarged surrounding lymph nodes enlargement (Fig. 2). An endoscopic ultrasound-guided fine-needle aspiration biopsy (EUS-FNAB) of tumor was performed (Fig. 3), and immunohistochemistry results CD34 ( −), c-kit ( −), desmin ( −), and S100 ( +) confirmed a benign esophageal schwannoma. He was referred to our department for surgical intervention. Considering the tumor size and location, we decided to perform enucleation of tumor using both thoracoscopic and cervical approaches. Under general anesthesia, the patient was first immobilized in the prone position, with port placements similar to those use of esophageal cancer procedures, specifically (1) air seal port at the right sixth intercostal space on subscapular angle, (2) 12-mm port at the right ninth intercostal space on scapular line, (3) 12-mm port at the right eighth intercostal space on mid-axilla line, (4) 5-mm port at the right seventh intercostal space on posterior-axilla line, (5) 5-mm port at the right fifth intercostal space on posterior-axilla line, and (6) 12-mm port at the right third intercostal space on mid-axilla line. No apparent invasion was observed after circumferential dissection of the caudal side of the tumor and its separation from surrounding organs (Fig. 4). The tumor extended to the upper thoracic esophagus. We performed dissection as much as possible toward the side of the cervical esophagus under thoracic procedures. After changing the patient’s position from prone to supine, a collar-shaped skin incision was performed on the neck. The cervical esophagus was identified from the left side, and after confirming the presence of the left recurrent laryngeal nerve, periesophageal dissection was performed. To acquire clear view of operation field, the right sternocleidomastoid muscle was dissected from the sternum. After confirming the right recurrent laryngeal nerve, the esophagus was taped above and below the tumor, and the muscular layer was incised to enucleate the tumor. After enucleate tumor, dissected muscular layer was sutured with 3–0 absorbable sutures; however, a 1-cm muscular layer defect remained. Intraoperative endoscopy suggested that further muscular layer suture would result in stenosis of the cervical esophagus. Therefore, we decided to perform a right sternocleidomastoid muscle flap to repair muscular layer and to prevent stenosis of the cervical esophagus (Fig. 5). The operation time was 4 h and 29 min (53 min for thoracic procedures), and blood loss was 60 ml. The excised specimen was 6 × 5.2 × 4.2 cm (Figs. 6 and 7). The postoperative course was uneventful, and fluoroscopy on postoperative day 8 confirmed no anastomotic leakage or stenosis. Oral intake was started on day 9. The patient had an uneventful recovery and was discharged in good condition on the 22nd postoperative day. There were no symptoms of stenosis after discharge and no recurrence 2 years after surgery (Fig. 8).

## Discussion and conclusions

Benign esophageal tumors account for less than 1% of all esophageal tumors. Among benign esophageal tumors, leiomyoma is the most common, and schwannoma is very rare [4]. The diagnostic accuracy of EUS-FNA for schwannomas is not yet high [5]. There is a report [6] that even benign schwannoma shows accumulation on FDG-PET scans, so it is difficult to make a diagnosis before surgery. A definitive diagnosis requires resection and immunohistochemical staining. Minimally invasive surgery for esophageal cancer has become common in Japan since the introduction of the thoracoscopic approach in the 1990s. On the other hand, the surgical technique for benign esophageal diseases still depends on the discretion of each institution. If the benign esophageal tumor is small, it can be followed up, but if it is symptomatic or suspected to be malignant, surgery is indicated. If the esophageal tumor is large, esophagectomy may be performed, but enucleation is often the procedure of choice. Esophageal schwannomas are often located in the upper thoracic esophagus region [7, 8]. The thoracic inlet is originally narrow in nature and surrounded by the clavicle, sternum, first rib, and thoracic vertebrae, and the mobility of the tumor is poor. It is generally difficult to operate on schwannomas arising in the cervicothoracic region using a single approach, such as the thoracic or the cervical approach only. Therefore, enucleation using both thoracoscopic and cervical approaches is a good indication for the treatment of schwannomas in the cervicothoracic border region.

We conducted a search on PubMed for “esophageal schwannoma” and found 17 cases [3, 6, 9–21], including our own, of tumors located from the cervical to upper thoracic esophagus between 2000 and 2020 (Table 1). Enucleation was performed in 15 cases, while subtotal esophagectomy was performed in 2 cases. The surgical approach varied, with eight cases using a cervical approach, six cases using a thoracic approach, two cases using a hybrid approach, and one case using an abdominocervical approach. Among the enucleation cases, eight involved total-layer resection, with five cases following EUS-FNA. Esophageal wall repair was done with two-layer sutures in six cases and simple mucosal sutures plus sternocleidomastoid muscle flaps in two cases. Regardless of tumor size(cranial-caudal), resection was successful using either the cervical or thoracic approach. Tumor location influenced the choice of surgical procedure, with a cervical approach favored if the cranial side of the tumor was less than 20 cm from the incisors and a thoracic approach preferred if the tumor’s caudal end exceeded 30 cm. In cases where the tumor was on the left side of the esophagus or had a transverse diameter exceeding 5 cm, thoracoscopic surgery was challenging and required careful consideration to prevent recurrent nerve damage. In our case, the tumor was located on the right side of the esophagus, making it suitable for thoracoscopic surgery. However, due to a narrow thoracic inlet and a tumor transverse diameter exceeding 5 cm, dissection of a cranial side of the tumor was difficult for thoracoscopy, and the recurrent nerve could not be confirmed. Preoperative EUS-FNA also influenced the potential for complete resection due to adhesion, necessitating consideration of esophageal wall repair. Although one case reported esophageal stricture, no other complications or recurrences were mentioned in the reports.

**Table 1:** Search on PubMed: case reports of esophageal schwannoma from the cervical to upper thoracic esophagus

No	Age	Sex	Location	Location from incisors (cm)	Tumor size (cranial-caudal) (cm)	Biopsy	FDG-PET	Operation	Approach	Mucosal defect	Repair	Pathology	Resected tumor size (cm)	Author	Year
1	54	F	CeUt	15–21	7	-	-	Subtotal esophagectomy	Abdominocervical	-	N/A	Malignant	6 × 6	Basoglu	2006
2	29	F	UtMtCe	19–27	8	-	-	Enucleation	Thoracotomy	+	Two-layer sutures	Benign	8 × 7.5 × 6	Mizuguchi	2007
3	22	M	UtCe	19–27	8	-	-	Enucleation	Thoracotomy	+	Two-layer sutures	Benign	8.5	Choo	2011
4	78	F	UtCe	N/A	2.8	-	7.8	Enucleation	Cervical	-	Simple suture	Benign	5 × 3 × 3	Nakatsu	2011
5	70	F	UtCe	17–20	3	-	6.8	Enucleation	Cervical	-	Simple suture	Benign	4 × 3.5 × 2.7	Nakatsu	2011
6	67	M	UtCe	20–25	5	-	10.85	Enucleation	Cervical, median sternotomy	-	Simple suture	Benign	6 × 9.5 × 3.7	Ojima	2013
7	69	F	CeUt	N/A	N/A	-	-	Enucleation	Cervical	-	Simple suture	Benign	5 × 2.3 × 2	Ferrante	2014
8	32	F	UtCe	20–29	9	+	9.8	Enucleation	Cervical	+	Two-layer sutures	Benign	8.7 × 5.9 × 2.4	Jeon	2014
9	37	F	UtCe	19-	N/A	+	-	Enucleation	Thoracotomy	-	Simple suture	Benign	3.5 × 3 × 3	Kozac	2015
10	62	F	CeUt	17–24	7	-	4.8	Enucleation	Thoracotomy	+	Two-layer sutures	Benign	9	Lui	2015
11	39	F	UtCe	19–24	5	+	5.5	Subtotal esophagectomy	Thoracoscopic	-	N/A	Benign	5.6 × 4.5 × 2.4	Watanabe	2016
12	36	F	CeUt	N/A	6.5	+	-	Enucleation	Cervical	+	Simple suture, sternocleidomastoid muscle flap	Benign	6.5 × 4.5	Ahn	2017
13	66	M	UtCe	25–30	5	+	-	Enucleation	Cervical	+	Two-layer sutures	Benign	5.2 × 4 × 3.1	Moro	2017
14	74	F	UtCe	23–28	5	-	15	Enucleation	Thoracotomy + cervical	-	Simple suture (stenosis +)	Benign	8 × 4.2	Iwata	2018
15	40	F	CeUt	N/A	4.6	+	-	Enucleation	Cervical	-	Simple suture	Benign	8 × 4.5 × 2	Emilio	2019
16	50	F	UtCe	20–30	10.5	-	-	Enucleation	Thoracotomy	+	Two-layer sutures	Benign	9.5 × 7 × 3	Jad	2019
17	58	M	CeUt	22–27	5	+	-	Enucleation	Thoracoscopic + cervical	+	Simple suture, sternocleidomastoid muscle flap	Benign	6 × 5.2 × 4.2	Our case	

*Legends*: *Ce* cervical esophagus, *Ut* upper thoracic esophagus, *N/A* not available

The thoracoscopic plus cervical approach has disadvantages such as a longer operative time and the need for repositioning of the patient. On the other hand, there are advantages of adding the cervical approach, for example, the direct visualization and palpation of the esophagus during resection and repair. In the present case, the mucosa could be repaired, but the defect in the muscular layer was large, and direct suture would have resulted in stenosis. In this case, we need an organ covering the defect. If we use a muscle valve in the thoracic cavity, it is necessary to dissect and cover the defect with a stemmed intercostal muscle valve. If the defect is large, the intercostal muscle valve may not be sufficient. In the cervical region, it is possible to use a muscle valve with the capacity of the sternocleidomastoid muscle as it is. The other advantage of using cervical approach is that we can confirm the degree of ischemia of the mucosa under direct vision. During operation, to acquire the esophagus above and below the tumor, the esophagus was dissected at least 8 cm circumferentially. The esophagus is fed by the tracheoesophageal artery, which branches from the inferior thyroid artery in the cervical esophagus, the bilateral bronchial arteries, and the proper esophageal artery in the thoracic esophagus. These vessels are anastomosed within the wall, and there is a well-developed vascular network in the submucosa [22]. Nevertheless, esophageal ischemia is a concern when the length of incised esophagus is large. In this case, since we were able to preserve both the azygos arch and the right bronchial artery, we could evaluate the color tone of the mucosa under direct observation to confirm that there was no problem regarding ischemia of the esophagus. Postoperative endoscopy revealed no stenosis or ischemic changes. Taken together, this experience indicated that our hybrid technique is a useful method that can safely resect even large benign esophageal tumors.

## Data Availability

Data sharing is not applicable to this article as no datasets were generated or analyzed during the current study.

## Abbreviations

EUS-FNAB: Endoscopic ultrasound-guided fine-needle aspiration biopsy
EGDs: Esophagogastroduodenoscopy
CT: Computed tomography
MRI: Magnetic resonance imaging
